# Impact of delay in the diagnosis and treatment of head and neck cancer^☆^^☆☆^

**DOI:** 10.1016/j.bjorl.2015.10.009

**Published:** 2015-11-06

**Authors:** André Wady Debes Felippu, Eduardo Cesar Freire, Ricardo de Arruda Silva, André Vicente Guimarães, Rogério Aparecido Dedivitis

**Affiliations:** a Department of Medicine, Faculdade de Ciências da Saúde, Universidade Metropolitana de Santos (UNIMES), Santos, SP, Brazil; b Department of Medicine, Universidade de São Paulo (USP), São Paulo, SP, Brazil; c Department of Otorhinolaryngology, Head and Neck Surgery, Universidade Metropolitana de Santos (UNIMES), Santos, SP, Brazil; d Department of Head and Neck Surgery, Hospital das Clínicas, Universidade de São Paulo (USP), São Paulo, SP, Brazil

**Keywords:** Delayed diagnosis, Head and neck neoplasms, Squamous cell carcinoma, Time factors, Primary health care, Prognosis, Diagnóstico tardio, Neoplasias de cabeça e pescoço, Carcinoma de células escamosas, Fatores de tempo, Atenção primária à saúde, Prognóstico

## Abstract

**Introduction:**

Head and neck tumors can be easily recognized through clinical evaluation. However, they are often diagnosed at advanced stages.

**Objective:**

To evaluate the delay from the patient's initial symptoms to the definitive treatment.

**Methods:**

Retrospective study of patients enrolled in 2011 and 2012. A questionnaire was filled in about socioeconomic aspects, patient history, tumor data, professionals who evaluated the patients, and the respective time delays.

**Results:**

The following time delay medians were observed: ten months between symptom onset and the first consultation; four weeks between the latter and the first consultation with a specialist; four weeks between the specialist consultation and diagnosis attainment; and 12 weeks between diagnosis and the start of treatment.

**Conclusions:**

Most head and neck tumors are diagnosed at advanced stages, due to patient and health care factors.

## Resumo

**Introdução:**

Apesar de poderem ser facilmente reconhecidos ao exame clínico, os tumores de cabeça e pescoço são, muitas vezes, diagnosticados em estadiamento avançado.

**Objetivo:**

Avaliar a demora entre o surgimento dos sintomas iniciais do paciente e seu encaminhamento para o tratamento definitivo.

**Método:**

Trata-se de um estudo retrospectivo de pacientes arrolados em 2011 e 2012. Foi preenchido questionário sobre fatores sócio-econômicos, antecedentes, dados do tumor, profissionais que avaliaram os pacientes e respectivos períodos de demora.

**Resultados:**

Foram observadas as seguintes medianas de tempo de demora: 10 meses entre o início dos sintomas e o primeiro atendimento; 4 semanas entre este e a primeira consulta com o especialista; 4 semanas entre esta e o estabelecimento do diagnóstico; e 12 semanas entre este e o início do tratamento.

**Conclusões:**

A maior parte dos cânceres de cabeça e pescoço é diagnosticada em estádios avançados, por fatores relacionados aos pacientes e à atenção à saúde.

## Introduction

Head and neck tumors are relatively easy to visualize or palpate at the clinical examination. Nevertheless, many patients are diagnosed at advanced stages of the disease, perhaps due to the lack of early alarming symptoms. This results in a lack of motivation to seek medical attention.^1^, ^2^

Head and neck cancers are often at an already advanced stage when diagnosed. The greater the delay in diagnosis and the onset of treatment, the more advanced the stage, the more aggressive the necessary therapy, and the worse the prognosis. This makes a fast and efficient diagnosis a challenge.^2^ Understanding the reasons responsible for late diagnosis of head and neck cancer could help to design interventions aimed at reducing the frequency of unfavorable outcomes.

If the time period between the initial consultation and treatment is prolonged, patients may experience tumor and clinical stage progression, which affects the therapeutic schedule with possible negative influence on prognosis. This is a relevant clinical problem, as comorbidity control prior to surgical treatment may require a long period.^3^

Lesion location and the different forms of tumor presentation and symptoms may contribute to the delay. Silent tumors, those with difficult access, or those that take longer to manifest obvious symptoms hinder the patient's perception, delaying the entire diagnostic process.^4^, ^5^ Therefore, very often, depending on the symptoms, it takes the patient longer to seek medical care.^6^ Studies have suggested increasing the awareness of individuals considered at risk, such as smokers and drinkers, to seek medical help after the initial symptoms, which favors prognosis.^7^ However, such delay may also be due to factors related to professional care and health care, with a time ranging in literature from four days to 3.5 months.^5^

The aim of this study is to assess the delay from symptom onset to the start of definitive treatment, and to identify any association between the delay and the patient's socio-economic status and tumor staging.

## Methods

This study was approved by the ethics committee of the institution where it was carried out, under No. 730.552. This is a retrospective study of new cases of squamous cell carcinoma of the upper aerodigestive tract, diagnosed at the Outpatient Clinic of Head and Neck Surgery of the institution, from January 2011 to August 2012. Tumors of the salivary glands and thyroid were excluded from this study, as they have different clinical presentation and biological behaviors from carcinoma of the upper aerodigestive tract.

Patients completed a questionnaire focused on epidemiological, disease, and treatment factors. Illiterate patients were aided by an accompanying family member and one of the authors was always available to resolve any doubts. The following data were collected: identification (name and registration number at the institution), age (in years), gender, ethnicity, educational level (from none to college/university), smoking and alcohol consumption (both measured semi-quantitatively) and primary tumor location. Tumors were staged according to the sixth edition of the TNM classification of the American Joint Committee on Cancer (AJCC). The professionals who treated the patient previously, as well as the main modality of cancer treatment the patient underwent (surgery, radiotherapy, or palliative chemotherapy) were also assessed.

The following durations were measured:

1. Interval between the reported symptom onset and seeking medical care (in months);
2. Interval between the first medical or dental appointment and the first consultation with the specialist (in weeks);
3. Interval between the first consultation with the specialist and the attainment of the diagnosis – anatomopathological results (in weeks);
4. Interval between diagnosis attainment and start of the treatment (in weeks).

## Results

Of the 80 patients identified, 75 were males (93.75%), and 63 were white (78.75%). Age ranged from 45 to 73 years, with a median of 63 years. Most of them regularly consumed tobacco products (98.75%) and alcohol (52.5%). As for educational level, most had not finished elementary school: 42 (52.5%). Among the tumor sites, the most frequent ones were oral cavity, 33 (41.84%), and larynx, 25 (31.25%). The distribution of the initial clinical stages showed a predominance of EC III, 23 (28.75%) and EC IV, 44 (55%) (Table 1).

**Table 1:** Epidemiological data of the assessed patients (*n* = 80).

	*n*	%
*Gender*		
Male	75	93.75
Female	5	6.25

*Ethnicity*		
White	63	78.75
Non-white	17	21.5

*Smoking*		
Regular smoker	79	98.75
Non-smoker	1	1.25

*Alcohol consumption*		
Regular user	42	52.5
Non-user	38	47.5

*Level of schooling*		
<Elementary school	42	52.5
Elementary school	32	40
<High school	3	3.75
High school	3	3.75

*Primary sites*		
Lips	2	2.5
Oral cavity	33	41.25
Oropharynx	8	10
Hypopharynx	5	6.25
Larynx	25	31.25
Paranasal sinuses	3	3.75
Primary occult	4	5

*Clinical staging*		
EC I	5	6.25
EC II	8	10
EC III	23	28.75
EC IV	44	55

*Main modality of referral*		
Surgery	23	28.75
Radiotherapy	52	65
Chemotherapy (palliative)	5	6.25

All 80 patients underwent a consultation with a non-specialist in head and neck surgery: 45 with an otorhinolaryngologist, 38 with a generalist, 12 with a dentist, and eight with other specialties, *i.e.*, three patients went to three different professionals before having a consultation with the expert, and 14 had a consultation with two different professionals beforehand.

The median time between symptom onset and seeking medical care was ten months; four months between the first general consultation motivated by the tumor and the first visit to the specialist; between the latter and diagnosis attainment, four months; and between the diagnosis attainment and start of the treatment, 12 months (with no difference between the main treatment modalities to which patients were referred to) (Table 2).

**Table 2:** Median and range of the intervals studied (*n* = 80).

Interval	Median	Range
Symptom onset – seeking health care service (months)	10	8–48
First consultation with a general practitioner – first consultation with a specialist (weeks)	4	2–180
First consultation with a specialist – establishing the diagnosis (weeks)	4	2–8
Diagnosis – start of the treatment (weeks)	12	2–16

## Discussion

There is a consensus that the prognosis of head and neck cancer at advanced stages (EC III and IV) is worse than that observed in tumors diagnosed in the early stages. Any delay in diagnosis and treatment can lead to a progression to more advanced stages, and thus a decrease in cure rates and treatment effectiveness.^3^ If the advanced disease is the result of the delay in case presentation or management, it can be concluded that the reduction of such interval would lead to treatment at an earlier stage, increasing survival rates and reducing morbidity.^4^

The delay in diagnosis refers to the total period of time between symptom onset and when the diagnosis is established. From then until the start of the treatment, the delay occurs in treatment planning and performance. One can also divide the delay into two phases: the period from symptom onset to the seeking of medical care (patient delay) and the excessive time between the first consultation with a health professional and the first consultation with a specialist (health system delay).^8^ This delay by the professional could also be defined as the time between the first medical consultation (usually with a non-specialist) and the date of the histopathological diagnosis. While professional delay can be measured relatively accurately, patient delay tends to show assessment error, as it depends on its degree and perception.^9^

It is believed that a longer period from symptom onset to diagnosis confirmation would explain the fact that the disease is diagnosed at advanced stages.^8^ Patient delay in seeking medical care can be due to factors related to the tumor, such as the primary tumor site and its characteristics, sometimes oligosymptomatic. Additionally, sociodemographic variables, such as socioeconomic status, can influence delay. Psychological factors must also be considered.^6^

In the present series, treated at a public outpatient clinic, the median time of symptom onset to medical consultation was ten months. This delay can be, in part, attributed to specific patient factors.^1^, ^2^ The population's difficulty in accessing a specialist seems to be due in part to their low socioeconomic status. Sometimes they lack funds even for the use of public transport. Patient delay, as mentioned before, is difficult to measure accurately because it is based on perception, which is highly subjective and can be influenced by many social and cultural factors.^9^ Additionally, when considering the entire case management, patients should also be carefully instructed about the return visits and follow-up.^1^

In the present study, the median time for a patient to be referred from primary care to a specialist (head and neck surgeon) was four weeks. One of the relatively quick factors was the fact that the initial evaluation was conducted by an otorhinolaryngologist, who recognized the situation and referred the patient to the cancer specialist.

However, it took some patients a long time to have a consultation with the head and neck surgeon, and in this case, the authors mainly recognize the lack of self-care on the part of most of these patients, who neglected their condition. However, other patients experienced a relative delay because of the professionals involved, as the patients had more than one consultation before the appointment with the specialist. Indeed, some general health care professionals failed to recognize certain signs and symptoms as suggestive of malignancy.

There are many problems involving delay in diagnosis, especially in the public health care system; there, a prompt initiation of an indicated treatment, including surgery, is not always possible. It is not only the intellectual and social status of the patient that impacts the difficulty in treating head and neck cancer, but also the many shortcomings of the public health care service, including referral, scheduling tests and treatment.

Studies have documented lower survival rates in populations with a poorer socioeconomic status.^10^ This reflects the occurrence of more advanced tumors in this group due, in part, to the lack of education (with limited use of preventive behaviors for health), delay in seeking medical care, and the reality of insufficient medical care.^9^ Even in cases of tumors whose location would facilitate the diagnosis – such as oral cancer – there is a tendency to delay, attributed to patient factors, as well as health professional factors–physicians and dentists.^11^ Much time is also lost waiting for the scheduling of more sophisticated imaging methods, often important for tumor staging, a crucial step to define the therapeutic strategy to be used.

After the anatomopathological diagnosis is established, the median time to the start of treatment was 12 weeks. This delay occurred irrespective of the main therapeutic modality for which the patient was referred (surgery, radiation therapy, or palliative chemotherapy), as it represents the current global reality of the public health care system patient in Brazil. This time includes the scheduling of the first consultation and treatment planning.

## Conclusion

Most head and neck malignant tumors are diagnosed at advanced stages. Delay occurred in all periods, both for diagnosis and treatment, due to both patient as well as health care factors.

## Conflicts of interest

The authors declare no conflicts of interest.
